# An analysis of the medial femoral condyle flap anatomy and the involvement of different tissue components for the reconstruction of complex defects

**DOI:** 10.1302/2046-3758.149.BJR-2024-0536.R2

**Published:** 2025-09-19

**Authors:** Michael Kohlhauser, Anna Vasilyeva, Heinz Bürger, Friedrich Anderhuber, Lars-Peter Kamolz, Michael Schintler

**Affiliations:** 1 Division of Plastic, Aesthetic and Reconstructive Surgery, Department of Surgery, Medical University of Graz, Graz, Austria; 2 Division of Hand Surgery, Private Hospital Maria Hilf, Klagenfurt am Woerthersee, Austria; 3 Institute for Macroscopic and Clinical Anatomy, Medical University of Graz, Graz, Austria; 4 COREMED – Centre for Regenerative Medicine and Precision Medicine, Joanneum Research, Graz, Austria

**Keywords:** Reconstructive surgery, Descending genicular artery, Medial femoral condyle flap, medial femoral condyle, artery, descending genicular artery, reconstructive surgery, femur, lower limbs, tendon, femoral artery, adductor magnus, vastus medialis muscle

## Abstract

**Aims:**

The reconstruction of complex defects involving various tissues still presents a challenge for reconstructive surgery and makes a combined flap indispensable. The mediodistal femur region (MDFR), which is supplied by the descending genicular artery (DGA), represents a unique donor site for harvesting combined flaps. This study analyzes the vascular anatomy of this region and the possible types of combined flaps.

**Methods:**

Within this analysis, the vascular supply of the DGA in a total of 35 lower limbs was investigated, having been embalmed with the Walter Thiel technique in order to enable lifelike conditions.

**Results:**

The DGA was detectable in 100% (n = 35) of all instances. The artery divided into three branches in 48.57% (n = 17) of cases and two branches in the remaining cases. In 40% (n = 14) of cases we found a saphenous artery (SA) and a musculoarticular branch (MAB), in 8.57% (n = 3) an articular branch (AB) and a muscular branch (MB), and in 2.86% (n = 1) a SA and a MB. Usage of DGA branches enabled corticoperiosteal, corticocancellous, osteochondral, or osteocutaneous flaps in 100% (n = 35) of our cases, and myocorticoperiostal, osteomyotendinous, osteomyotendocutanous, or osteotendofasciocutaneous flaps in 97.14% (n = 34). Vascular supply of skin flaps was feasible via the SA in 100% (n = 35) of cases or via dermal branches of the AB in 37.14% (n = 13).

**Conclusion:**

The multitissue, distal-mediofemoral region, supplied by the DGA and its branches, offers an optimal donor site with reliable vascularization, enabling the harvesting of combined flaps.

Cite this article: *Bone Joint Res* 2025;14(9):795–804.

## Article focus

- Our analysis investigated the vascular variations in the multitissue mediodistal femur region (MDFR), with particular emphasis on descending genicular artery (DGA) and its branches.
- Additionally, the possibility of different combined flaps supplied by branches of the DGA was analyzed.

## Key messages

- DGA-supplied tissues reliably offer the possibility to create combined flaps, which can then be used to treat complex tissue defects.

## Strengths and limitations

- In our study, all specimens were prepared using the Walter Thiel technique, creating lifelike conditions.
- We acknowledge the limitations of our analysis only as a representative sample of the global population.

## Introduction

Complex tissue defects continue to represent major challenges within reconstructive surgery. Combined flaps, consisting of either similar or dissimilar tissues, are indispensable in achieving success with functionality and tissue homeostasis restoration.^1^

The mediodistal femur region (MDFR), with long vascular pedicles^2,3^ and minimal donor site morbidity,^4,5^ enables combined-tissue flap harvesting with multiple applications. In 1981, Acland et al^6^ first described the use of neurovascular free flaps supplied by the saphenous artery (SA), a branch of the DGA. This flap, which may contain fascia and skin, can be employed for wound defect reconstruction with neural innervation via the saphenous or medial femoral nerve.^6,7^ The use of vascularized bone and tendon from the MDFR was first proposed by Masquelet et al^8^ in 1985. Additionally, Masquelet’s team investigated the usage of vascularized periosteal and osteoperiosteal flaps from the MDFR, with the further possibility of harvesting musculo-osteoperiosteal flaps to treat nonunion and segmental bone defects.^9-12^ These flaps were initially used for skull and nonunion reconstruction of upper limb long bones.^13-18^ Skin flaps can be simultaneously transferred with vascularized bone as osteocutaneous flaps.^13,19^ Recently, corticoperiosteal, corticocancellous, or osteochondral flaps from the medial femoral condyle (MFC) have gained popularity following successful carpal reconstruction results.^19-27^

The aim of this study was to analyze the vascular anatomy of the MDFR to evaluate the feasibility of various flaps and investigate their vascular supply.

## Methods

### Cadaveric specimens

Within this analysis, a total of 35 lower limbs were investigated. All body donors provided informed consent for the participation in scientific investigations and were part of the body donation programme of the Institute for Macroscopic and Clinical Anatomy at the Medical University of Graz.

### Embalming of the cadavers

Embalming of the cadavers was performed according to the technique developed by the anatomist Walter Thiel.^28^ This method allows embalmed cadavers to have a similar appearance, tissue colour, and consistency to fresh ones, with no change in textural characteristics of vessels, and thus their diameter can be precisely measured.^28,29^

### Flap harvesting procedure

All dissections were performed with cadavers in supine position, having external rotation of leg and hip, and knee joint flexure. A slightly curved incision is made from the MFC to mid-thigh, along the medial edge of the vastus medialis muscle (VMM). The visualization of the DGA and its branches is generally performed in distal to proximal direction. After splitting the VMM fascia, the muscle is retracted ventrally, allowing dissection up to the periosteum.

Firstly, the articular branch (AB) of the DGA is located between the tendon of the adductor magnus (ToAM) and the medial edge of the VMM. Secondly, the DGA is identified from distal to proximal towards the adductor canal to its origin from the femoral artery (FA). Thirdly, we identified the other DGA-derived branches, whose muscular branch (MB), the SA, and the accompanying saphenous nerve (SN) penetrate the aponeurosis of the adductor canal. Their pathways between the gracilis and the sartorius muscles (SM) are dissected until all branches are uncovered.

### Vascular analysis method

All measures were conducted under loupe glasses magnification. DGA branches were identified and each respective vessel was measured lengthways with a ruler from its origin, while diameters were measured with calipers. For accuracy, the whole analysis was performed by two independent investigators (AV, MS).

### Statistical analysis

The evaluation of data was carried out using descriptive analysis, which was conducted using SPSS v29 (IBM, USA).

## Results

**Fig. 1:**
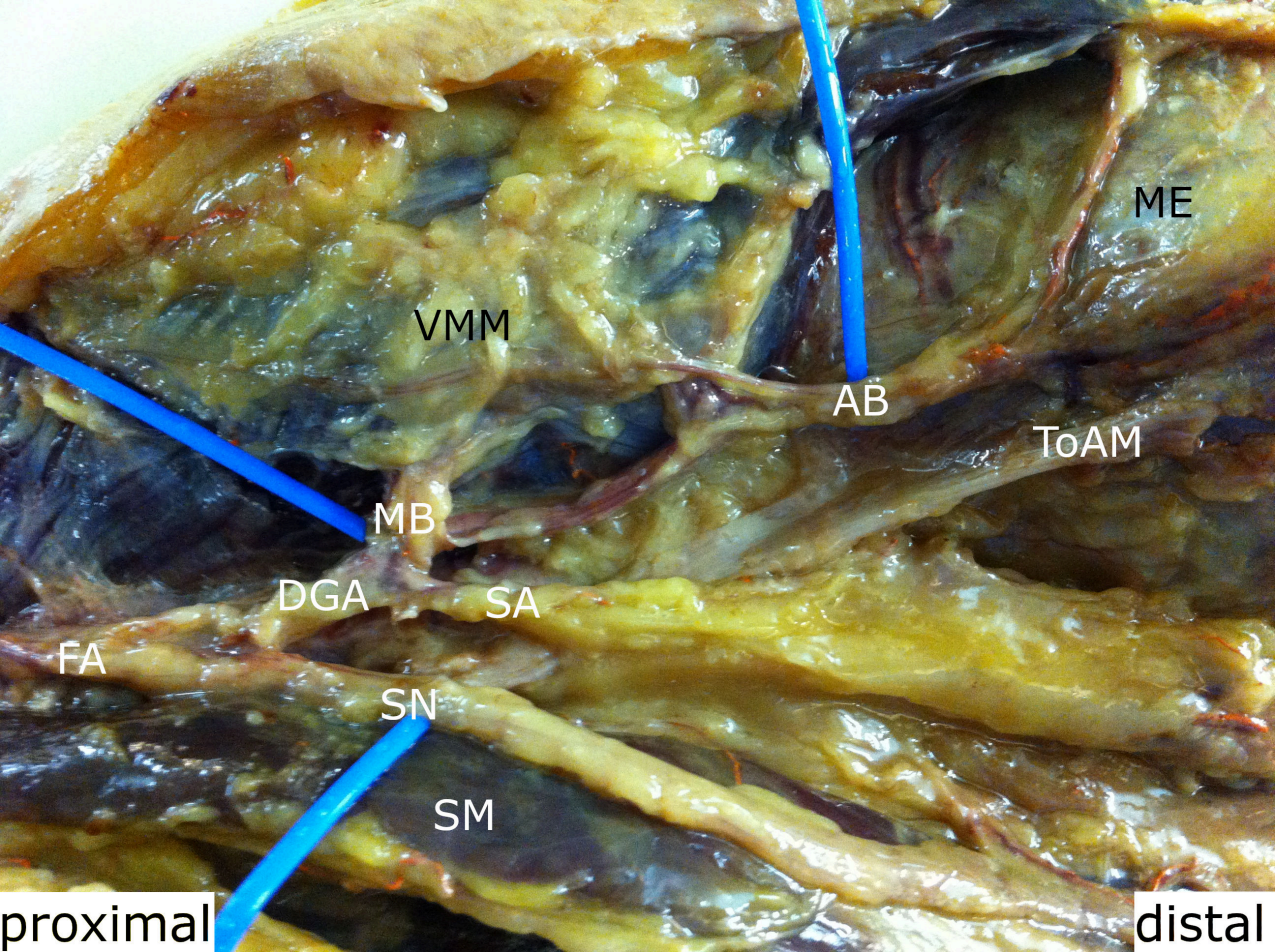
Proximal to the medial epicondylus (ME), the descending genicular artery divided into the saphenous artery (SA), an articular branch (AB), and a muscular branch (MB). FA, femoral artery; SM, sartorius muscle; SN, saphenous nerve; ToAM, tendon of the adductor magnus; VMM, vastus medialis muscle.

**Fig. 2:**
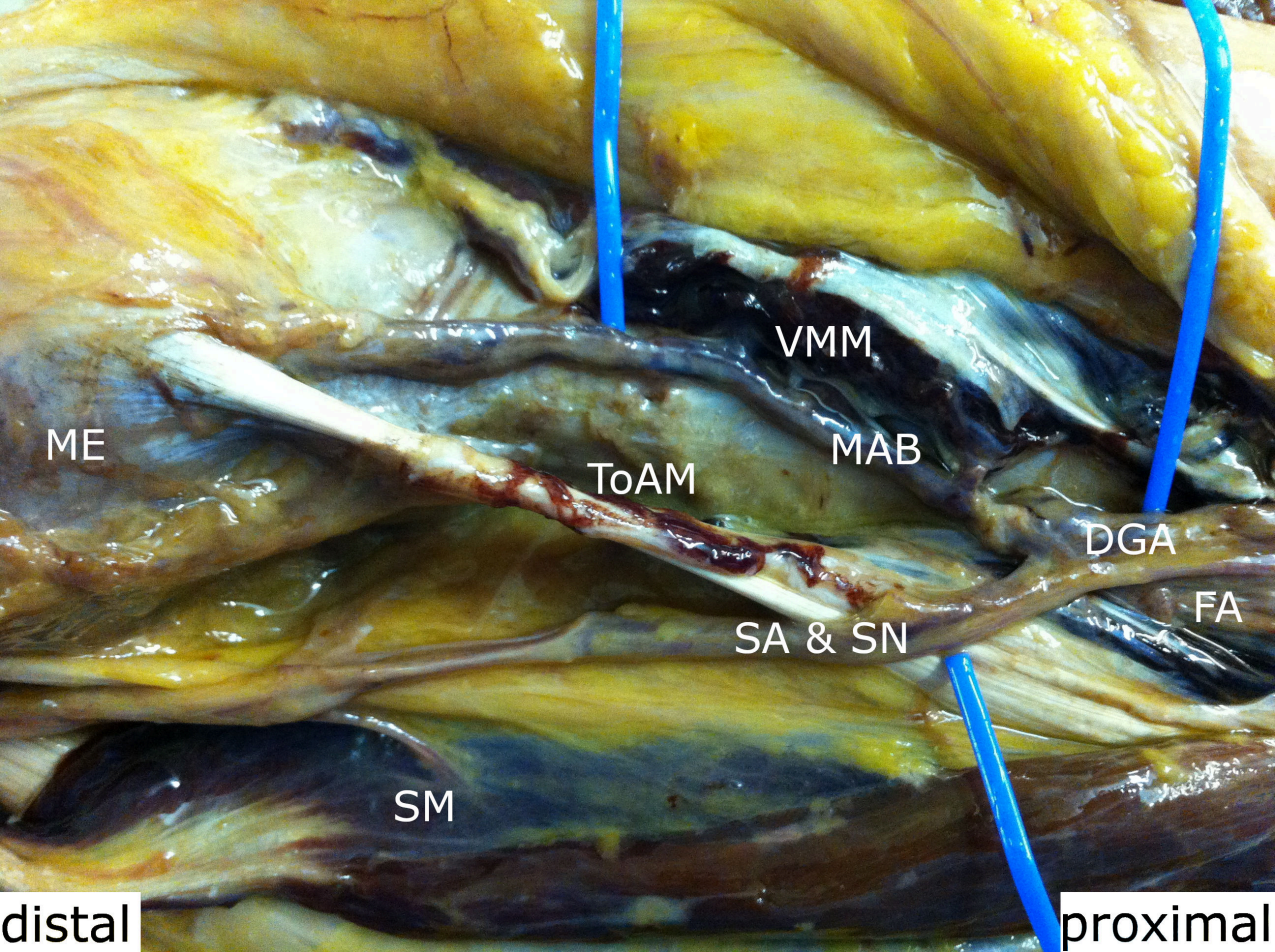
The descending genicular artery divided into a saphenous artery (SA) and a musculoarticular branch (MAB). FA, femoral artery; ME, medial epicondylus; SM, sartorius muscle; SN, saphenous nerve; ToAM, tendon of the adductor magnus; VMM, vastus medialis muscle.

**Fig. 3:**
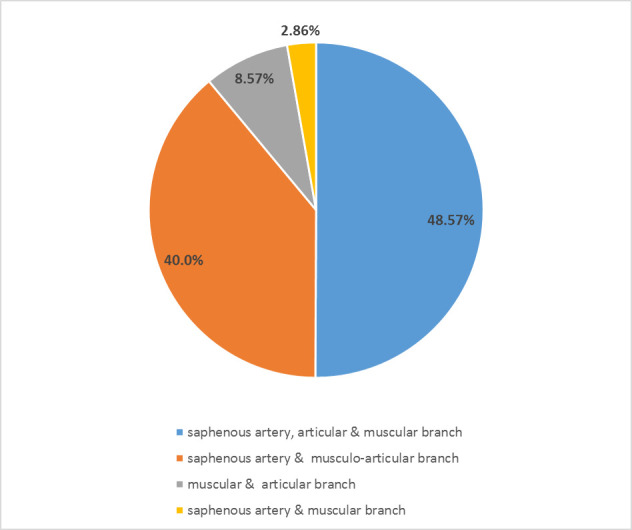
The percentage allocation of the different descending genicular artery branching patterns.

In all our cases, the DGA originated from the FA at a mean of 14.3 cm (11.5 to 19.5) from the knee joint line, within the region of the adductor hiatus. Roughly 2.1 cm (0.5 to 7.0) distal from origin, the DGA, with a mean diameter of 1.9 mm (1.0 to 2.5), divided into further branches. In 48.57% (n = 17) of instances, the SA, an AB, and a MB were present (Figure 1). In 51.43% (n = 18) of the instances, two branches existed, being either a SA and a musculoarticular branch (MAB) (40%, n = 14) (Figure 2), an AB and a MB (8.57%, n = 3), or less frequently, a SA and a MB (2.86%, n = 1). Each arterial branch was accompanied by veins. Figure 3 illustrates the percentage distribution of the DGA’s various branching patterns.

**Fig. 4:**
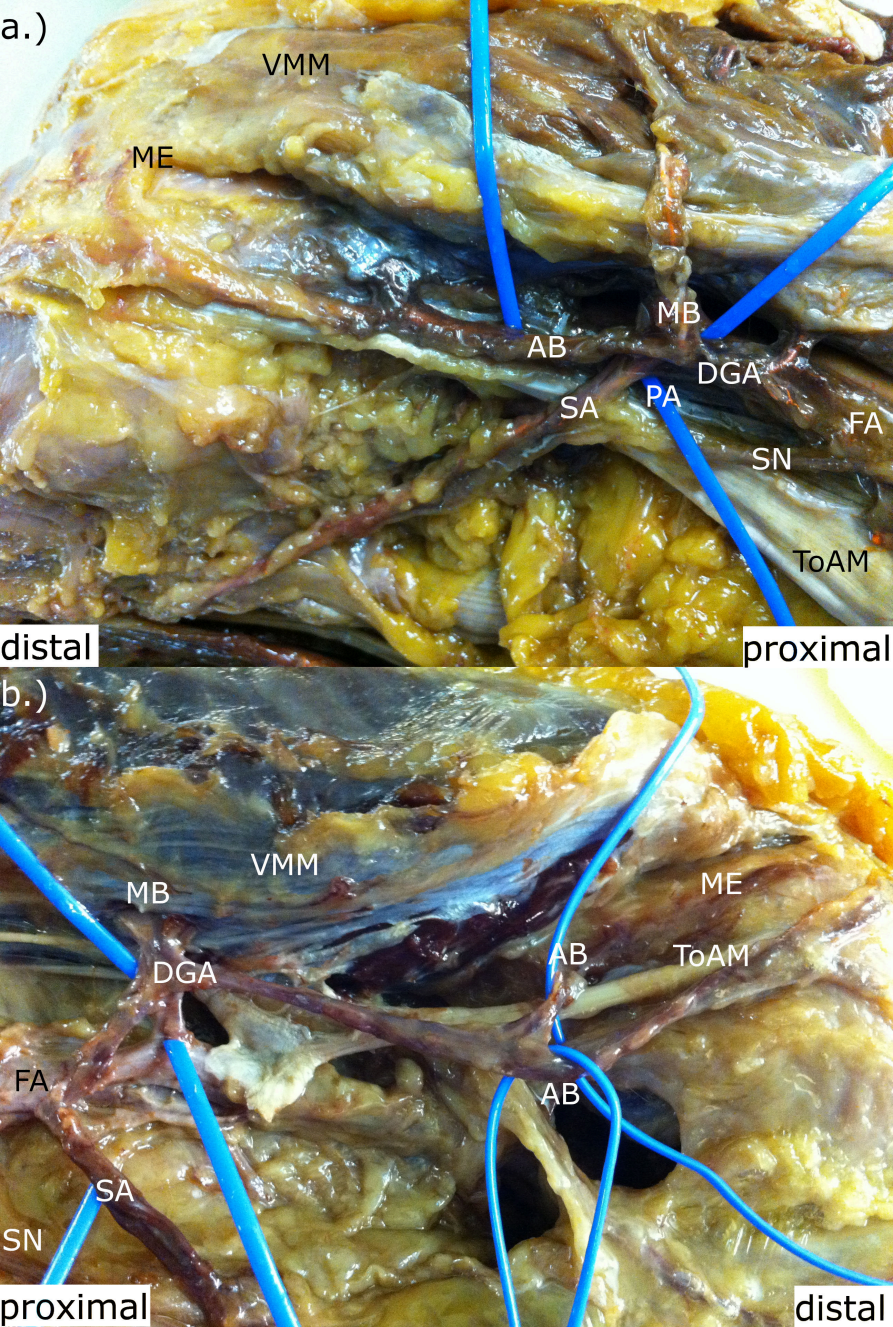
a) The origin of the saphenous artery (SA) from the popliteal artery (PA), and b) from the femoral artery (FA). AB, articular branch; DGA, descending genicular artery; MB, muscular branch; ME, medial epicondylus; SM, sartorius muscle; SN, saphenous nerve; ToAM, tendon of the adductor magnus; VMM, vastus medialis muscle.

On average, SA length was 13.91 cm (10.5 to 23.5) and diameter 1.00 mm (0.50 to 2.00). It originated in 8.57% (n = 3) of all cases from the popliteal artery (PA) (5.71%, n = 2) or FA (2.86%, n = 1), instead of the DGA (Figure 4). In 60% (n = 21) of all cases, the MB originates from the DGA, with a mean length of 1.67 cm (1.0 to 3.5) and diameter of 1.1 mm (0.5 to 2.0), supplying the VMM with several small branches.

**Fig. 5:**
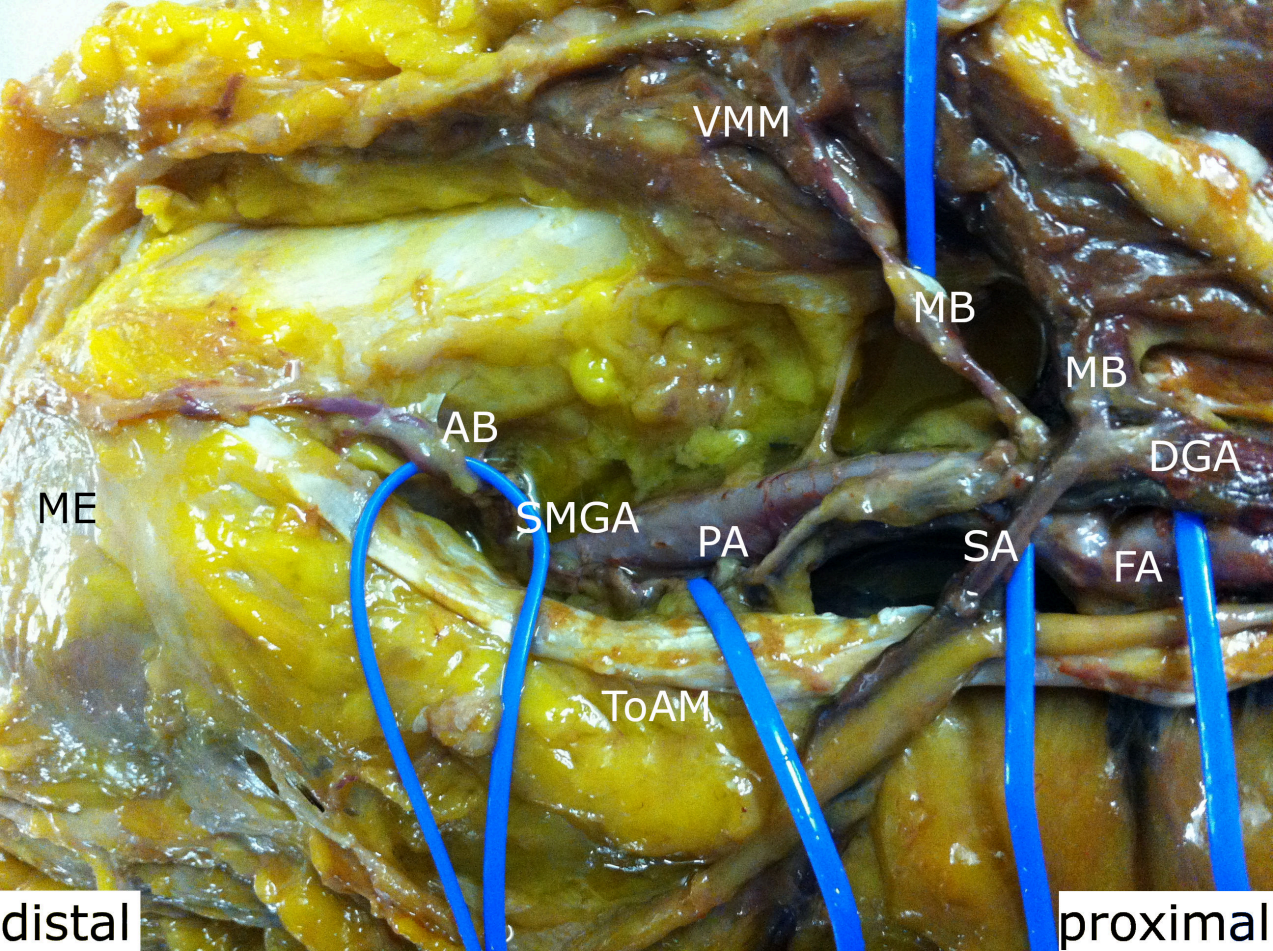
The articular branch (AB) origin from the superior medial genicular artery (SMGA) instead of the descending genicular artery (DGA). FA, femoral artery; MB, muscular branch; ME, medial epicondylus; ToAM, tendon of the adductor magnus; VMM, vastus medialis muscle.

**Fig. 6:**
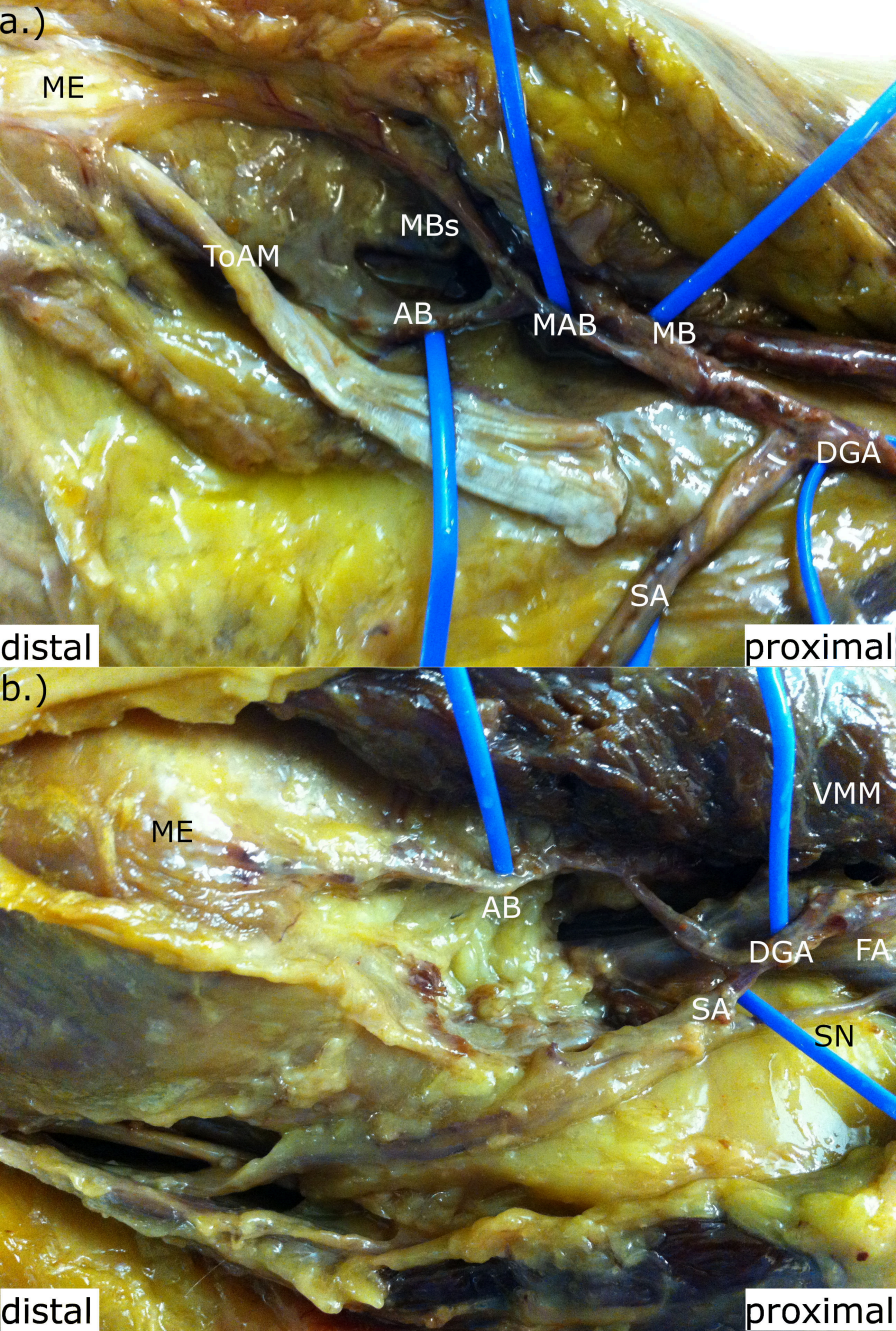
a) The descending genicular artery (DGA) divided into a saphenous artery (SA), a muscular branch (MB) and an articular branch (AB), bifurcating into further MBs. b) The DGA divided into a SA and an AB, with the latter branching into several muscular branches. FA, femoral artery; ME, medial epicondylus; SN, saphenous nerve; ToAM, tendon of the adductor magnus; VMM, vastus medialis muscle.

In 97.14% (n = 34) of instances, the AB originated from the DGA, and, in 2.86% (n = 1), separately from the superior medial genicular artery (SMGA) (Figure 5). With an average length of 6.76 cm (5.0 to 11.0) and diameter of 1.1 mm (0.5 to 1.5), the AB extended on the posterior surface of the medial intermuscular septum, along the ToAM to the MFC, supplying its periosteum and underlying bone with terminal branches. Upon descending, the AB released smaller branches that vascularize the ToAM. In all cases, the AB divided at the MFC into a longitudinal and transverse branch, which respectively supplied the periosteum and underlying bone, and the bone and cartilage of the femoral patellar surface. In 80% (n = 28) of all cases, the AB supplied the VMM with approximately two muscular branches (1 to 4), achieving this in 40% (n = 14), even when a MB originated from the DGA (Figure 6a). However, in 40% (n = 14) of cases, the MB was absent and the distal VMM was solely supplied by the AB of the DGA instead, resulting in the formation of a MAB (Figure 6b). Only 20% (n = 7) of our cases exhibited a unique vascular supply of the distal VMM through the MB of the DGA.

In light of these findings, DGA-vascularized muscle, periosteum, bone, and cartilage may be obtained in 100% (n = 35) of cases. Since in 2.86% (n = 1) of all cases the AB originated from the SMGA, vascularized tendon and myocorticoperiostal or osteomyotendinous flaps from DGA branches were only possible in 97.14% (n = 34).

**Fig. 7:**
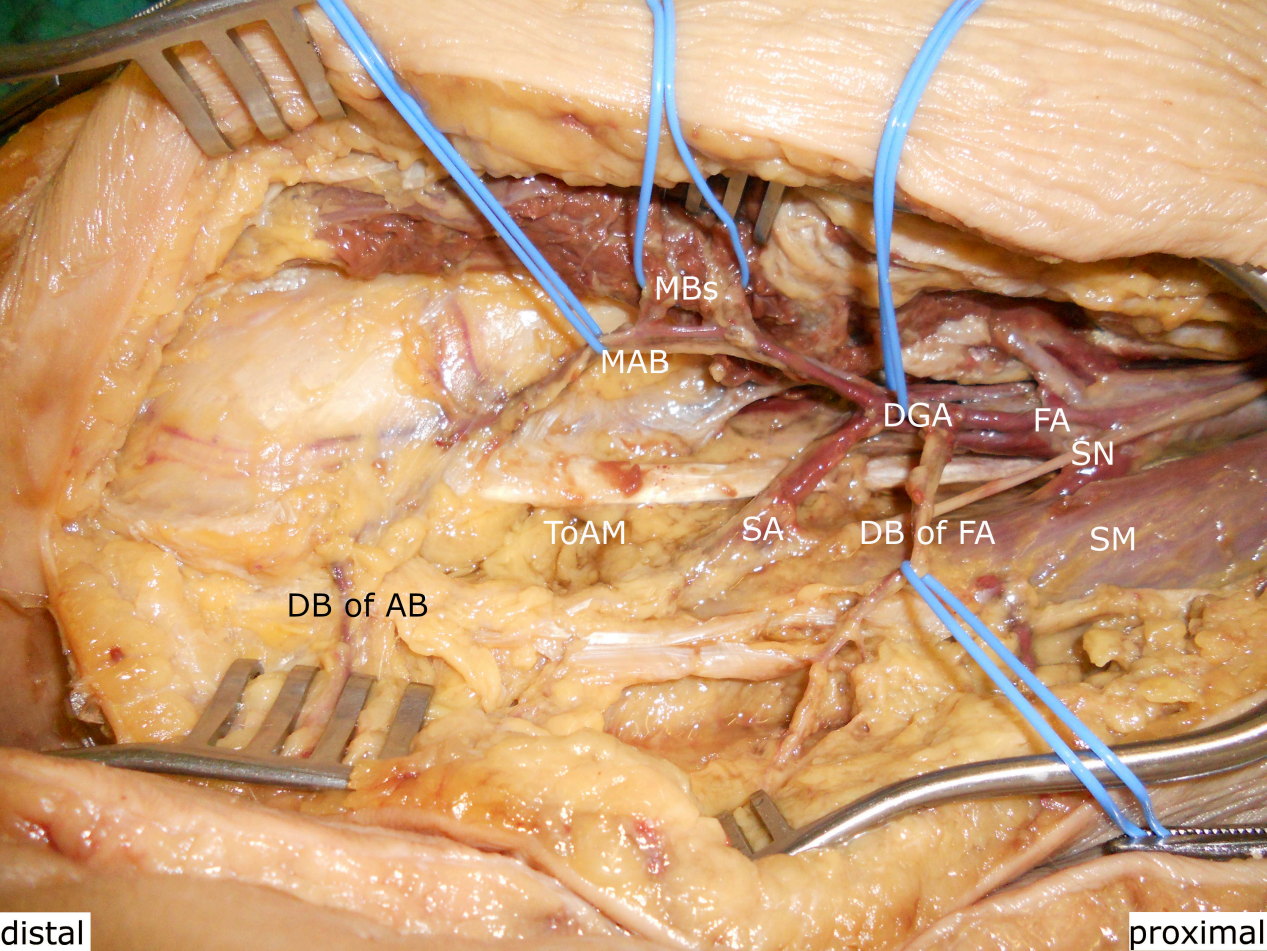
The articular branch (AB) divided into dermal branches (DBs) to supply the overlying skin. DGA, descending genicular artery; FA, femoral artery; MAB, musculoarticular branch; MB, muscular branch; ME, medial epicondylus; SA, saphenous artery; SN, saphenous nerve; ToAM, tendon of the adductor magnus.

**Fig. 8:**
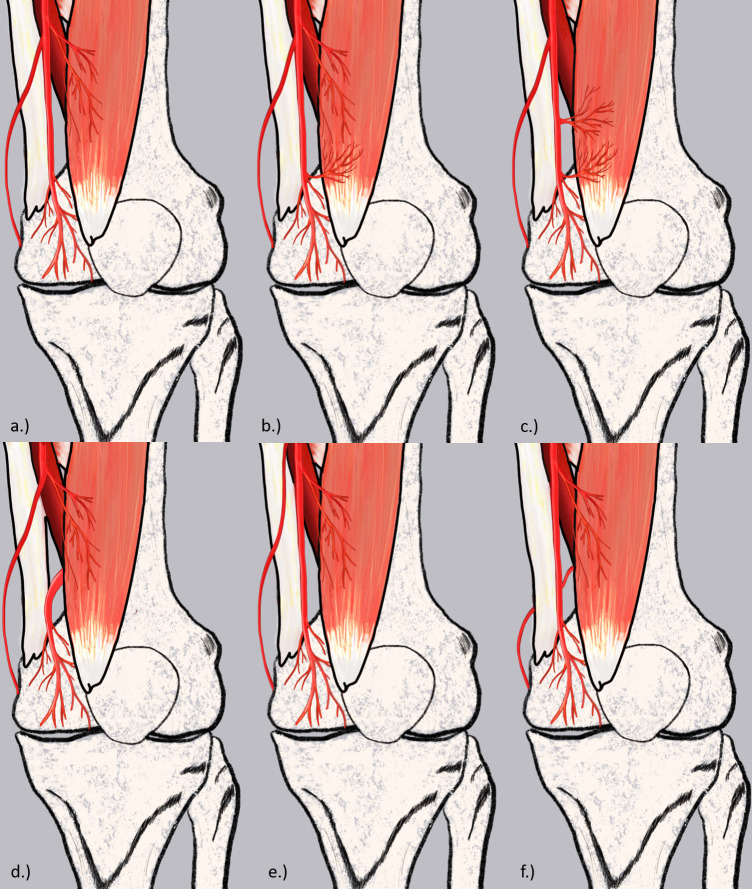
Schematic representation of the various vascular variations identified. a) Descending genicular artery (DGA) divided into saphenous artery (SA), articular branch (AB), and muscular branch (MB). b) DGA divided into SA, musculoarticular branch (MAB), and MB. c) DGA divided into SA and MAB. d) DGA divided into SA and MB, AB originated from superior medial genicular artery (SMGA). e) DGA divided into AB and MB, SA originated from femoral artery (FA). f) DGA divided into AB and MB, SA originated from popliteal artery (PA).

In 37.14% (n = 13) of all cases, the AB divides into dermal branches (DBs), enabling the dissection of skin flaps (Figure 7). In 8.57% (n = 3) of cases where the SA did not originate from the DGA, DBs bifurcated from the AB. Consequently, skin flap harvesting from the DGA was possible in 100% (n = 35) of cases. Furthermore, osteotomy-tendocutanous or osteotendofasciocutaneous flaps supplied by DGA branches could be obtained in 97.14% (n = 34) of all cases. Figure 8 shows a schematic overview of the different variations identified.

In all cases, accompanying veins, terminating in the femoral vein, were identified. Furthermore, venous outflow from large skin flaps in the mid lower third of the thigh was possible using saphenous vein branches, present in 100% (n = 35) of cases. This region’s sensory innervation was proximally provided by the anterior femoral cutaneous nerve and obturator nerve, and distally by the SN.

**Fig. 9:**
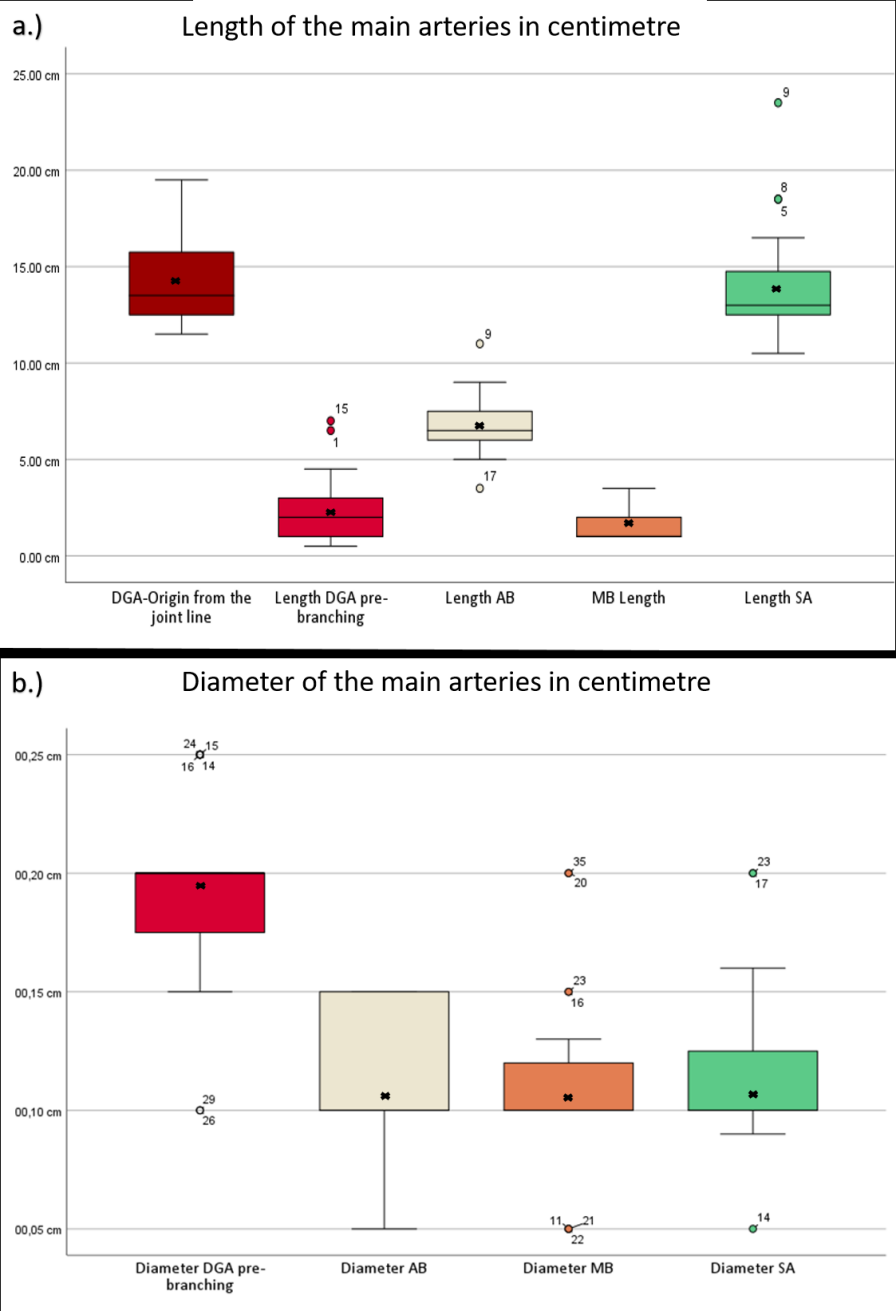
Boxplot diagrams showing a) length and b) diameter of descending genicular artery (DGA) and its branches. X = average, median = line. Outliers are displayed as points. MB, muscular branch; SA, saphenous artery.

Table I shows different specimen data, while Table II and Table III present the descriptive statistics. Figure 9 illustrates boxplot diagrams relating to vessel length and diameter.

**Table I.** Data from analysis of vascular structures in the mediodistal femur region of each individual anatomical specimen.

Study sample	DGA - origin from the joint line, cm	Length DGA pre-branching, cm	Diameter DGA, cm	Length AB, cm	Diameter AB, cm	AB originates from DGA	Muscle-supplying branches originate from AB (number)	Dermal branches originate from the articular branch	A transverse branch originates from AB	MB originates from DGA	Length MB, cm	Diameter MB, cm	SA originates from DGA	SA originates from the PA	Length SA, cm	Diameter SA, cm
P1	19.00	6.50	0.20	8.00	0.15	Yes	3	No	Yes	No	-	-	Yes	No	16.50	0.15
P2	17.00	4.00	0.15	7.00	0.10	Yes	2	Yes	Yes	No	-	-	Yes	No	14.50	0.10
P3	15.50	1.00	0.20	6.50	0.10	Yes	1	Yes	Yes	Yes	1.00	0.10	Yes	No	15.50	0.10
P4	12.00	2.00	0.25	5.50	0.15	Yes	2	Yes	Yes	Yes	1.00	0.10	No	Yes	13.50	0.10
P5	17.00	3.00	0.20	8.50	0.10	Yes	1	No	Yes	No	-	-	Yes	No	18.50	0.10
P6	11.50	0.50	0.20	7.00	0.15	Yes	1	Yes	Yes	Yes	1.00	0.10	Yes	No	10.50	0.10
P7	12.00	2.00	0.15	5.00	0.05	Yes	2	No	Yes	No	-	-	Yes	No	16.50	0.10
P8	12.00	2.50	0.25	6.00	0.10	Yes	2	No	Yes	No	-	-	Yes	No	18.50	0.10
P9	18.00	3.00	0.20	11.00	0.10	Yes	2	No	Yes	No	-	-	Yes	No	23.50	0.10
P10	14.50	2.50	0.15	5.00	0.05	Yes	0	Yes	Yes	Yes	1.00	0.10	Yes	No	12.50	0.10
P11	12.00	1.50	0.15	8.00	0.15	Yes	1	Yes	Yes	Yes	3.50	0.05	No	Yes	15.00	0.10
P12	13.00	0.50	0.15	6.50	0.09	Yes	0	No	Yes	Yes	1.00	0.12	Yes	No	13.00	0.10
P13	15.00	4.00	0.20	6.50	0.10	Yes	1	Yes	Yes	Yes	3.00	0.13	Yes	No	14.00	0.15
P14	16.00	0.50	0.25	9.00	0.05	Yes	4	Yes	Yes	Yes	1.50	0.10	Yes	No	12.00	0.05
P15	19.00	7.00	0.25	7.00	0.15	Yes	0	No	Yes	Yes	2.50	0.10	Yes	No	15.00	0.10
P16	19.50	4.50	0.25	6.00	0.10	Yes	0	No	Yes	Yes	3.50	0.15	Yes	No	12.00	0.10
P17	15.00	3.00	0.20	3.50	0.15	No*	0	No	Yes	Yes	3.00	0.10	Yes	No	14.00	0.20
P18	13.00	2.00	0.15	6.00	0.10	Yes	3	No	Yes	No	-	-	Yes	No	12.00	0.10
P19	13.00	2.00	0.15	6.00	0.10	Yes	3	No	Yes	No	-	-	Yes	No	12.50	0.10
P20	16.00	2.00	0.20	7.00	0.10	Yes	2	No	Yes	Yes	2.00	0.20	Yes	No	11.50	0.15
P21	12.50	0.50	0.20	5.50	0.15	Yes	1	No	Yes	Yes	1.00	0.05	Yes	No	13.00	0.10
P22	13.50	1.50	0.20	8.00	0.10	Yes	1	Yes	Yes	Yes	1.50	0.05	Yes	No	12.00	0.15
P23	12.50	1.00	0.20	6.00	0.15	Yes	0	Yes	Yes	Yes	1.00	0.15	No	No	12.50	0.20
P24	14.50	1.00	0.25	6.50	0.10	Yes	1	No	Yes	Yes	1.50	0.10	Yes	No	11.50	0.10
P25	12.00	1.50	0.20	5.50	0.10	Yes	0	No	Yes	Yes	1.00	0.10	Yes	No	11.00	0.15
P26	12.00	1.00	0.10	5.50	0.09	Yes	2	No	Yes	No	-	-	Yes	No	14.00	0.09
P27	12.00	0.50	0.20	6.00	0.10	Yes	2	No	Yes	No	-	-	Yes	No	14.00	0.10
P28	12.50	0.50	0.20	6.50	0.10	Yes	2	No	Yes	No	-	-	Yes	No	13.00	0.10
P29	17.00	2.00	0.10	9.00	0.10	Yes	2	No	Yes	No	-	-	Yes	No	16.00	0.10
P30	13.00	0.50	0.20	8.00	0.10	Yes	1	Yes	Yes	No	-	-	Yes	No	14.00	0.10
P31	12.50	1.50	0.20	7.00	0.10	Yes	1	No	Yes	Yes	1.00	0.10	Yes	No	12.50	0.10
P32	14.00	1.00	0.20	7.50	0.05	Yes	1	No	Yes	Yes	1.00	0.10	Yes	No	13.00	0.10
P33	13.00	1.00	0.20	7.00	0.10	Yes	2	Yes	Yes	Yes	2.00	0.10	Yes	No	13.00	0.10
P34	15.50	3.00	0.20	6.00	0.15	Yes	2	Yes	Yes	No	-	-	Yes	No	13.00	0.16
P35	15.50	3.00	0.20	7.50	0.15	Yes	2	No	Yes	Yes	1.00	0.20	Yes	No	13.50	0.15

* AB was present, but it originated from the SMGA rather than from the DGA.

AB, articular branch; DGA, descending genicular artery; MB, muscular branch; PA, popliteal artery; SA, saphenous artery; SMGA, superior medial genicular artery.

**Table II.** Percentage distribution of vascular observations.

Vascular observation	Percentage
AB originated from DGA	97.14
AB originated from SMGA	2.86
Muscle-supplying branches originate from AB	80
Dermal branches originate from AB	37.14
MB originated from DGA	60
SA originated from DGA	91.43
SA originated from AP	5.71
SA originated from FA	2.86

DGA, descending genicular artery; FA, femoral artery; MB, muscular branch; SA, saphenous artery; SMGA, superior medial genicular artery.

**Table III.** Data on arterial branch dimensions.

Arterial branch dimensions	Median (IQR)	Mean (SD)	Range
DGA - origin from the joint line, cm	13.50 (12.50 to 16.00)	14.34 (2.33)	11.5 to 19.5
Length DGA pre-branching, cm	2.00 (1.00 to 3.00)	2.10 (1.60)	0.5 to 7.0
Diameter DGA, cm	0.20 (0.15 to 0.20)	0.19 (0.04)	0.10 to 0.25
Length AB, cm	6.50 (6.00 to 7.50)	6.76 (1.39)	5.0 to 11.0
Diameter AB, cm	0.10 (0.10 to 0.15)	0.11 (0.03)	0.05 to 0.15
Muscle-supplying branches originate from AB, n	1.00 (1.00 to 2.00)	1.43 (1.01)	1 to 4
Length MB, cm	1.00 (1.00 to 2.25)	1.67 (0.89)	1.0 to 3.5
Diameter MB, cm	0.10 (0.10 to 0.13)	0.11 (0.04)	0.05 to 0.20
Length SA, cm	13.00 (12.5 to 15.0)	13.91 (2.52)	10.5 to 23.5
Diameter SA, cm	0.10 (0.10 to 0.15)	0.11 (0.03)	0.05 to 0.20

DGA, descending genicular artery; MB, muscular branch; SA, saphenous artery.

## Discussion

Complex defects associated with a loss of various tissue components require replacement through the use of combined flaps for stable wound healing. Anatomical analyses are indispensable in investigating potential donor regions for possible combined flaps. In this context, we have vascularly analyzed the mediodistal thigh region for the possibilities of combined flaps.

For this analysis, we used cadaver specimens, prepared using the Walter Thiel embalming technique.^28^ This method creates lifelike conditions and is used in microsurgical vascular research.^29,30^ Therefore, we expect our analysis to reflect near-realistic conditions.

When dissecting the DGA and its branches, anatomical variations must be considered. In some cases, the DGA may be absent, and the SA, MB, and AB arise from the FA and/or PA. Hertel and Masquelet^9^ observed the DGA in 92% of all instances, and Weitgasser et al^31^ in 98%. Larson et al^27^ and Yamamoto et al^32^ reported the occurrence of this artery in 89%. As in Rahmanian-Schwarz et al,^33^ the DGA was observed in all specimens in our analysis.

The DGA can bifurcate into either three or two branches in a range of variations, as in 51.43% (n = 18) of cases in our study. These variations include a SA with a MAB or MB, or a MB with an AB. In the latter, the SA originates directly from the FA or PA, or may be absent.

In 91.43% (n = 32) of cases studied, the SA originated from the DGA, in 5.71% (n = 2) from the PA, and in 2.86% (n = 1) from the FA. According to Rahmanian-Schwarz et al,^33^ the SA originated from the DGA in 71% of cases, while Hertel and Masquelet^9^ reported a 64% incidence. In 80% of cases in Weitgasser et al,^31^ the SA originated from the DGA, while 18% were from the superficial FA. However, in the final 2%, the DGA was absent and a prominent SMGA was discovered, while the SA was absent.

Conversely, in Iorio et al,^34^ the SA originated from the DGA in 58.33% of cases, in 25% simultaneously with the DGA from the superficial FA, in 8.33% independently of the DGA from the superficial FA, and was absent in 8.33%.

In both Larson et al^27^ and Yamamoto et al,^32^ a SA originated from the DGA in only 79% of cases. The SA can be used to obtain both a fasciocutaneous^6^ and an osteocutaneous flap.^13,18,35^ The advantage is that the SA DBs may supply a larger skin area than DBs from the AB.^35^ In addition to wound closure, these skin flaps allow postoperative control of blood flow within the vascularized bone graft, regardless of the supplying DBs.^13,18,35^

Another application involves incorporating SA-supplied subcutaneous fat for soft-tissue reconstruction, as reported by Gaggl et al.^36^

ABs were absent in 2.4% of cases in Masquelet et al,^8^ 4% in Hertel and Masquelet,^9^ 10% in Larson et al,^27^ and 11% in Yamamoto et al.^32^ Hertel and Masquelet^9^ reported that the AB originated directly from the FA in 6% of cases. Our analysis showed an AB originating from the DGA in 97.14% (n = 34) of all cases and from the SMGA in only 2.86% (n = 1). Furthermore, the AB divided into one to four muscular branches in 80% (n = 28) of all instances, thus forming the MAB.

A MB from the DGA was observed in 60% (n = 21) of cases in our study, as opposed to 84% in Hertel and Masquelet.^9^ The distal VMM received vascular supply solely from the MB in 20% (n = 7) of cases, from the AB concurrently in 40% (n = 14), and exclusively from the latter in 40% (n = 14).

The VMM belongs to the type II classification of muscle flaps by Mathes and Nahai.^37^ The superficial FA provides the dominant blood supply with the DGA branches additionally supplying the distal part. A few cases in the literature report a myo-osseus or an osteomyocutaneous flap nourished by a muscle-supplying DGA branch: this technique has been applied in the reconstruction of calcaneous,^33^ tibial,^38^ and femoral bone defects.^39^ In 97.14% (n = 34) of our cases, myo-osseus or osteomyocutaneous flaps, supplied by the DGA branches, were possible. In 2.86% (n = 1), the AB originated from the SMGA and can be used for osteochondral flaps instead of the DGA, resulting in shorter pedicles.

Additionally, supply of the ToAM by DGA-derived AB permits the harvesting of a vascularized tendon. Masquelet et al^8^ decribed the transfer of vascularized ToAM for reconstruction of knee joint ligaments. Another application is Achilles tendon reconstruction, combined with skin as a tendocutaneous flap.^40-42^ Neuwirth et al^43^ used free vascularized ToAM in reconstruction of hand and foot extensor tendons, as tendinous, tendofasciocutaneous, or osteotendofasciocutaneous flaps.

We identified DBs originating from the AB in 37.14% (n = 13) of all cases, while Martin et al^13^ reported such branches in 86%. These branches are significantly advantageous when small skin pedicles are required, as their preparation is more efficient than SA branches. In contrast, the use of SA-derived pedicles enables larger flap movement-radius. Furthermore, these AB-derived DBs represent alternative vascular sources for combined flaps when the SA is absent, or does not originate from the DGA.

**Fig. 10:**
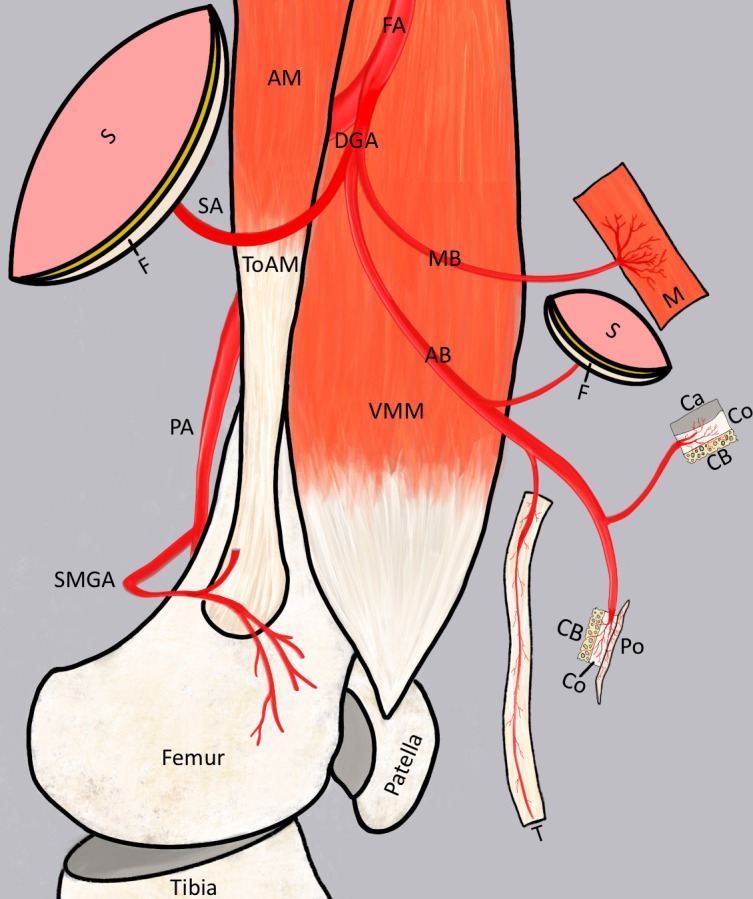
Schematic image of the descending genicular artery (DGA)-supplied tissues for flap combinations: skin (S), fascia (F), muscle (M), cartilage (Ca), cortical bone (CB), cancellous bone (CB), periosteum (Po), and tendon (T). AB, articular branch; AM, adductor magnus; FA, femoral artery; MB, muscular branch; SMGA, superior medial genicular artery; ToAM, tendon of the adductor magnus; VMM, vastus medialis muscle.

To summarize, the usage of DGA branches enables corticoperiosteal, corticocancellous, osteochondral, or osteocutaneous flaps in 100% (n = 35) of our cases and myocorticoperiostal, osteomyotendinous, osteomyotendocutanous, or osteotendofasciocutaneous flaps in 97.14% (n = 34). Vascular supply of skin flaps was feasible via the SA in 100% (n = 35) of cases or via DBs of the AB in 37.14% (n = 13). Figure 10 illustrates various flap options through the usage of DGA-supplied tissues.

Through this anatomical analysis, we aimed to illustrate the potential combined flaps options enabled by the unique anatomy of the MDFR. As demonstrated in this analysis, the combination of vascularized bone with cartilage, periosteum, tendon, muscle, fascia, and skin is feasible. This holds relevance for the reconstruction of articular surfaces and bone defects, including nonunions, following trauma and infections. The described flap techniques facilitate the restoration of small articular surfaces and bony structures, including the adjacent skin or tendon.

However, a restriction is posed by injuries or previous surgeries in the MDFR region. Another limitation for the vascularized bone graft is the size of the bony or cartilaginous defects; it is constrained by the dimensions of the non-weightbearing area of the MFC, which varies depending on body size, and has been used by members of our team at a maximum size of 6 × 4 × 0.4 cm as a thin corticoperiosteal bone graft, or 1 × 1.5 × 1.8 cm as osteochondral flap. Nevertheless, in a common application area such as scaphoid reconstruction, an average size of 1 × 1 × 1 cm is adequate. The size and shape of the MFC flap, particularly in peripheral regions such as the hand and foot, provide crucial advantages over conventional vascularized bone grafts, such as those derived from the pelvis or fibula. These can only be combined with skin and, due to their size, form, and pedicle, cannot always be optimally adjusted. Moreover, the conventional vascularized bone grafts are not suitable for articular surface reconstruction.

All the various flap combinations described in this analysis have been successfully employed by members of our team within reconstructive surgery. A clinical analysis with over ten years of practical experience, from 2013 to the present, is in progress and will follow this cadaveric study.

In summary, complex tissue defects associated with a loss of various tissues represent particular challenges in reconstructive surgery. The MDFR, supplied by the DGA and SGMA branches, offers an optimal donor site with reliable vascularization. This region permits combined flap harvesting consisting of various tissue components. In most of our cases, we demonstrated that harvesting of vascularized skin, fascia, bone, cartilage, tendon, or muscle to form combined flaps was possible. Thus, our analysis highlights the specific role of the MDFR, particularly its vascular supply, via the DGA in reconstructive surgery. In conclusion, this anatomical analysis serves as the foundation for raising awareness about the potential combined flap options enabled by the unique anatomy of the MDFR, particularly for the reconstruction of complex defects which affect several tissue components.

## Data Availability

All data generated or analyzed during this study are included in the published article and/or in the supplementary material

## References

[1] Hallock GG The complete nomenclature for combined perforator flaps Plast Reconstr Surg 2011 127 4 1720 1729 10.1097/PRS.0b013e31820a662b 21460679

[2] Ziegler T Kamolz LP Vasilyeva A Schintler M Neuwirth M Parvizi D Descending genicular artery. Branching patterns and measuring parameters: a systematic review and meta-analysis of several anatomical studies J Plast Reconstr Aesthet Surg 2018 71 7 967 975 10.1016/j.bjps.2018.03.005 29655665

[3] Sighary M Sajan A Walsh J Márquez S Cadaveric classification of the genicular arteries, with implications for the interventional radiologist J Vasc Interv Radiol 2022 33 4 437 444 10.1016/j.jvir.2021.12.019 34952196

[4] Rao SS Sexton CC Higgins JP Medial femoral condyle flap donor-site morbidity: a radiographic assessment Plast Reconstr Surg 2013 131 3 357e 362e 10.1097/PRS.0b013e31827c6f38 23446585

[5] Windhofer C Wong VW Larcher L Paryavi E Bürger HK Higgins JP Knee donor site morbidity following harvest of medial femoral trochlea osteochondral flaps for carpal reconstruction J Hand Surg Am 2016 41 5 610 614 10.1016/j.jhsa.2016.01.015 26948187

[6] Acland RD Schusterman M Godina M Eder E Taylor GI Carlisle I The saphenous neurovascular free flap Plast Reconstr Surg 1981 67 6 763 774 10.1097/00006534-198106000-00009 7243977

[7] Guang-xiang H Tong-bo Z Fa-bin W et al. Medial flap of the shank – anatomical study and clinical application J Tongji Med Univ 1986 6 4 246 250 10.1007/BF02909753 3806744

[8] Masquelet AC Nordin JY Guinot A Vascularized transfer of the adductor magnus tendon and its osseous insertion: a preliminary report J Reconstr Microsurg 1985 1 3 169 176 10.1055/s-2007-1007071 3903149

[9] Hertel R Masquelet AC The reverse flow medial knee osteoperiosteal flap for skeletal reconstruction of the leg. Description and anatomical basis Surg Radiol Anat 1989 11 4 257 262 10.1007/BF02098691 2617406

[10] Romana MC Masquelet AC Vascularized periosteum associated with cancellous bone graft: an experimental study Plast Reconstr Surg 1990 85 4 587 592 10.1097/00006534-199004000-00014 2315398

[11] Masquelet AC Romana MC Carlioz H Vascularized periosteal grafts. Anatomic description, experimental study, preliminary report of clinical experience Rev Chir Orthop Reparatrice Appar Mot 1988 74 Suppl 2 240 243 3231789

[12] Penteado C Masquelet A Romana M Chevrel J Anatomical bases of medical, radiological and surgical techniques periosteal flaps: anatomical bases of sites of elevation Radiol Anat J Clin Anat 1990 12 3 7 10.1007/BF02094118 2345893

[13] Martin D Bitonti-Grillo C De Biscop J et al. Mandibular reconstruction using a free vascularised osteocutaneous flap from the internal condyle of the femur Br J Plast Surg 1991 44 6 397 402 10.1016/0007-1226(91)90194-o 1933107

[14] Lapierre F Masquelet A Aesch B Romana C Goga D Cranioplasties using free femoral osteo-periostal flaps Chirurgie 1991 117 4 293 296 1817825

[15] Kobayashi S Kakibuchi M Masuda T Ohmori K Use of vascularized corticoperiosteal flap from the femur for reconstruction of the orbit Ann Plast Surg 1994 33 4 351 357 10.1097/00000637-199410000-00001 7810950

[16] Sakai K Doi K Kawai S Free vascularized thin corticoperiosteal graft Plast Reconstr Surg 1991 87 2 290 298 10.1097/00006534-199102000-00011 1989022

[17] Doi K Sakai K Vascularized periosteal bone graft from the supracondylar region of the femur Microsurgery 1994 15 5 305 315 10.1002/micr.1920150505 7934797

[18] Muramatsu K Doi K Ihara K Shigetomi M Kawai S Recalcitrant posttraumatic nonunion of the humerus: 23 patients reconstructed with vascularized bone graft Acta Orthop Scand 2003 74 1 95 97 10.1080/00016470310013734 12635801

[19] Bürger HK Windhofer C Gaggl AJ Higgins JP Vascularized medial femoral trochlea osteocartilaginous flap reconstruction of proximal pole scaphoid nonunions J Hand Surg Am 2013 38 4 690 700 10.1016/j.jhsa.2013.01.036 23474156

[20] Bürger HK Windhofer C Gaggl AJ Higgins JP Vascularized medial femoral trochlea osteochondral flap reconstruction of advanced Kienböck disease J Hand Surg Am 2014 39 7 1313 1322 10.1016/j.jhsa.2014.03.040 24855965

[21] Jones DB Bürger H Bishop AT Shin AY Treatment of scaphoid waist nonunions with an avascular proximal pole and carpal collapse. Surgical technique J Bone Joint Surg Am 2009 91 Suppl 2 169 183 10.2106/JBJS.I.00444 19805581

[22] Jones DB Bürger H Bishop AT Shin AY Treatment of scaphoid waist nonunions with an avascular proximal pole and carpal collapse. A comparison of two vascularized bone grafts J Bone Joint Surg Am 2008 90-A 12 2616 2625 10.2106/JBJS.G.01503 19047706

[23] Doi K Oda T Soo-Heong T Nanda V Free vascularized bone graft for nonunion of the scaphoid J Hand Surg 2000 25 3 507 519 10.1053/jhsu.2000.5993 10811756

[24] Pet MA Assi PE Yousaf IS Giladi AM Higgins JP Outcomes of the medial femoral trochlea osteochondral free flap for proximal scaphoid reconstruction J Hand Surg Am 2020 45 4 317 326 10.1016/j.jhsa.2019.08.008 31629563

[25] Pet MA Assi PE Giladi AM Higgins JP Preliminary clinical, radiographic, and patient-reported outcomes of the medial femoral trochlea osteochondral free flap for lunate reconstruction in advanced Kienböck disease J Hand Surg Am 2020 45 8 774 10.1016/j.jhsa.2019.12.008 32147088

[26] Van Handel AC Lynch LM Daruwalla JH Higgins JP Allen KL Pet MA Medial femoral trochlea flap reconstruction versus proximal row carpectomy for Kienböck’s disease: a morphometric comparison J Hand Surg Eur Vol 2021 46 10 1042 1048 10.1177/17531934211031862 34289733

[27] Larson AN Bishop AT Shin AY Free medial femoral condyle bone grafting for scaphoid nonunions with humpback deformity and proximal pole avascular necrosis Tech Hand Up Extrem Surg 2007 11 4 246 258 10.1097/bth.0b013e3180cab17c 18090830

[28] Thiel W Supplement to the conservation of an entire cadaver according to W. Thiel Ann Anat 2002 184 3 267 269 10.1016/s0940-9602(02)80121-2 12061344

[29] Wolff K-D Kesting MR Mücke T Rau A Hölzle F Thiel embalming technique: a valuable method for microvascular exercise and teaching of flap raising Microsurgery 2008 28 4 273 278 10.1002/micr.20484 18383351

[30] Odobescu A Moubayed SP Harris PG Bou-Merhi J Daniels E Danino MA A new microsurgical research model using Thiel-embalmed arteries and comparison of two suture techniques J Plast Reconstr Aesthet Surg 2014 67 3 389 395 10.1016/j.bjps.2013.12.026 24507964

[31] Weitgasser L Cotofana S Winkler M et al. Detailed vascular anatomy of the medial femoral condyle and the significance of its use as a free flap J Plast Reconstr Aesthet Surg 2016 69 12 1683 1689 10.1016/j.bjps.2016.09.024 27793561

[32] Yamamoto H Jones DB Moran SL Bishop AT Shin AY The arterial anatomy of the medial femoral condyle and its clinical implications J Hand Surg Eur Vol 2010 35 7 569 574 10.1177/1753193410364484 20237188

[33] Rahmanian-Schwarz A Spetzler V Amr A Pfau M Schaller HE Hirt B A composite osteomusculocutaneous free flap from the medial femoral condyle for reconstruction of complex defects J Reconstr Microsurg 2011 27 4 251 260 10.1055/s-0031-1275489 21424991

[34] Iorio ML Masden DL Higgins JP Cutaneous angiosome territory of the medial femoral condyle osteocutaneous flap J Hand Surg Am 2012 37 5 1033 1041 10.1016/j.jhsa.2012.02.033 22483181

[35] Hsu C-C Tseng J Lin Y-T Chimeric medial femoral condyle osteocutaneous flap for reconstruction of multiple metacarpal defects J Hand Surg Am 2018 43 8 781 10.1016/j.jhsa.2018.03.025 29735291

[36] Gaggl A Bürger H Chiari FM The microvascular osteocutaneous femur transplant for covering combined alveolar ridge and floor of the mouth defects: preliminary report J Reconstr Microsurg 2008 24 3 169 175 10.1055/s-2008-1076753 18454356

[37] Mathes SJ Nahai F Classification of the vascular anatomy of muscles: experimental and clinical correlation Plast Reconstr Surg 1981 67 2 177 187 7465666

[38] Kruger EA Ben-Amotz O Mendenhall SD Levin LS The chimeric myo-osseous medial femoral condyle flap for tibial nonunion: a case report Eplasty 2018 18 e23 30159107 PMC6053650

[39] Polykandriotis E Stangl R Hennig HH et al. The composite vastus medialis-patellar complex osseomuscular flap as a salvage procedure after complex trauma of the knee--an anatomical study and clinical application Br J Plast Surg 2005 58 5 646 651 10.1016/j.bjps.2005.01.008 15925343

[40] Gao J Xu D Wu S Transplantation of the vascularized adductor magnus tendon for the repair of Achilles tendon defect Chinese J Orthop 1999 19 656 658

[41] Huang D Wang HW Xu DC Wang HG Wu WZ Zhang HR An anatomic and clinical study of the adductor magnus tendon-descending genicular artery bone flap Clin Anat 2011 24 1 77 83 10.1002/ca.21060 20890971

[42] Sananpanich K Kraisarin J Descending genicular artery free flaps: multi-purpose tissue transfers in limb reconstruction J Plast Reconstr Aesthet Surg 2015 68 6 846 852 10.1016/j.bjps.2015.02.003 25837160

[43] Neuwirth M Bürger H Palle W Rab M One-stage reconstruction of isolated and combined tendon defects with the vascularized adductor magnus tendon graft: surgical technique and preliminary results J Plast Reconstr Aesthet Surg 2016 69 7 928 935 10.1016/j.bjps.2016.02.014 27056634

